# The Syntaxin-1A gene single nucleotide polymorphism rs4717806 associates with the risk of ischemic heart disease

**DOI:** 10.1097/MD.0000000000015846

**Published:** 2019-06-14

**Authors:** Franca R. Guerini, Enrico Ripamonti, Andrea S. Costa, Milena Zanzottera, Cristina Agliardi, Elisabetta Bolognesi, Mario Clerici, Vittorio Racca

**Affiliations:** a IRCCS Fondazione Don Carlo Gnocchi, Milano; b Pathophysiology and Transplantation, University of Milano, Milano, Italy.

**Keywords:** coronary artery disease, ischemic heart disease, Syntaxin-1A, insulin resistance

## Abstract

Ischemic heart disease (IHD) has a genetic predisposition and a number of cardiovascular risk factors are known to be affected by genetic factors. Development of metabolic syndrome and insulin resistance, strongly influenced by lifestyle and environmental factors, frequently occur in subjects with a genetic susceptibility. The definition of genetic factors influencing disease susceptibility would allow to identify individuals at higher risk and thus needing to be closely monitored.

To this end, we focused on a complex of soluble-N-ethylmaleimide-sensitive factor attachment protein receptors (SNAREs), playing an important role in metabolic syndrome and insulin resistance, involved in endothelial dysfunction and heart disease. We assessed if genetic variants of the SNARE genes are associated with IHD.

*SNAP25 rs363050*, *Stx-1A rs4717806*, *rs2293489*, and *VAMP2 26bp ins/del* genetic polymorphisms were analyzed in a cohort of 100 participants who underwent heart surgery; 56 of them were affected by IHD, while 44 were not. A statistical association of plasma glycemia and insulin resistance, calculated as Triglyceride glucose (TyG) index, was observed in IHD (*P* < .001 and *P* = .03, respectively) after binomial logistic stepwise regression analysis, adjusted by age, gender, diabetes positivity, waist circumference, and cholesterol plasma level. Among genetic polymorphisms, *rs4717806(A)* and *rs2293489(T)*, as well as the *rs4717806 – rs2293489 (A-T)* haplotype were associated with higher risk for IHD (*Pc* = .02; *Pc* = .02; *P* = .04, respectively). Finally, a statistical association of *rs4717806(AA)* genotype with higher TyG index in IHD patients (*P* = .03) was highlighted by multiple regression analysis considering log-transformed biochemical parameters as dependent variable and presence of coronary artery disease, age, gender, waist circumference, presence of diabetes as predictors. These results point to a role of the *Stx-1A rs4717806* SNP in IHD, possibly due to its influence on Stx-1A expression and, as a consequence, on insulin secretion and glucose metabolism.

## 1 Introduction

Ischemic heart disease (IHD), characterized by atherosclerotic coronary artery lesions, has a well-known genetic predisposition and tends to cluster in families.^[1]^ A number of cardiovascular risk factors that recognize a familiar aggregation have been identified; these include serum cholesterol, blood pressure levels, diabetes, and obesity.^[2]^ Environmental influence such as air pollution, as well as modifiable lifestyle habits, including diet, low levels of physical activity and smoke, also contribute to the modulation of IHD risk.^[3]^ The occurrence of IHD, nevertheless, cannot be explained by the variation of the traditional cardiovascular risk influences alone, and the development of risk charts based on them would misclassify a high proportion of cases, suggesting that other, still unknown, genetic elements play an important role in this condition.^[4]^ The role of genetics in the etiology of coronary artery disease is only partially understood and there is a great need to clarify the genetic basis of susceptibility to IHD, in order to identify cases with a true heritable component of this condition.

Epidemiological studies indicate that IHD is associated with the metabolic syndrome,^[5–7]^ defined as a cluster of glucose intolerance, hypertension, dyslipidemia, and central obesity. In particular, a low response to insulin action in adipose tissue, skeletal muscle, and liver, resulting in insulin resistance, is known as the headstream of metabolic syndrome.

A recent study highlighted the central role played by a complex of soluble-N-ethylmaleimide-sensitive factor attachment protein receptors (SNAREs) in metabolic diseases,^[8,9]^ which are also involved in the pathogenesis of type 2 diabetes mellitus (T2DM)^[10]^ as well as in cardiac functions.^[11–13]^ The SNARE-complex includes the two t-SNARE proteins, synaptosomal protein of 25 kDa (SNAP25) and syntaxin 1A (Stx-1A), as well as the v-SNARE protein VAMP2. To allow exocytosis the amino terminal of SNAP25 binds to Stx-1A and the carboxy-terminal binds to VAMP2, forming the four-helical bundle that brings secretory granules in close contact with the plasma membrane, thus enabling fusion to occur.

Stx-1A protein, in particular, is widely expressed in the brain, in the endocrine system and in the heart.^[14]^ This protein was shown to regulate signaling pathways in myocardial ischemic reperfusion injury, such as K_ATP_ channels and calcium channels.^[14–17]^ Notably, Stx-1A undergoes up-regulation as a consequence of ischemia,^[18]^ suggesting a potential role of Stx-1A in cardiac injury.

Two specific singular nucleotide polymorphisms (SNP), namely *rs4717806* and *rs2293489*, located, respectively in *Stx-1A-* locus in intron 9 and in *WBSCR22* gene, upstream to 5′UTR, are suspected to be involved in protein expression.^[19,20]^

SNAP25, on the other hand, is known to modulate several processes besides the actual fusion event, including the activity of potassium voltage gated (Kv) 2.1 channels.^[21]^ A specific SNP *rs363050* located into intron 1 of the *SNAP25* gene has been associated with SNAP25 protein expression.^[22]^ Finally, a *VAMP2* gene deletion of 16bp located at 2 kb from 3′ flanking regions of human *VAMP2*, in an intergenic region^[23]^ was associated with neurologic disease,^[24]^ and is suspected to impair SNARE functionality, indicating the need to investigate this deletion, as well as mechanisms involving SNARE activity.

We evaluated the association of the over described genetic non coding variants of the SNARE complex genes, suggested to be involved in protein expression,^[19,20,22,23]^ with IHD to ascertain a possible involvement of the SNARE complex in the risk to develop IHD. We also defined possible correlations between genetic SNARE polymorphism and biological pattern at risk for IHD. To this end, the distribution of *SNAP25 rs363050, Stx-1A rs4717806, rs2293489*, and *VAMP2 26bp ins/del* genetic polymorphisms were analyzed in a clinically and biochemically characterized cohort of patients who had recently undergone heart surgery.

## 2 Materials and methods

### 2.1 Patients

We prospectively and consecutively enrolled 100 adult patients (61 males and 39 females, all Caucasians), aged >18 years (mean age = 71 years; SD = 10.7), admitted as in-patients to the Cardiology Rehabilitation Department of the Don Carlo Gnocchi Foundation (Milan), after undergoing elective heart surgery.

Heart surgery interventions included coronary artery by-pass grafting (CABG), valve replacement or repair and/or ascending aorta surgery. Patients after heart transplant or left ventricular assist device implant were excluded. The study has been conducted in accordance with the declaration of Helsinki and the research protocol was approved by the Ethical Committee of the Don Carlo Gnocchi Foundation (protocol number: 16/2015/CE_FdG/SA). Informed consent to participate in the study has been signed by all participants.

Venous blood sample was collected at the beginning of the rehabilitation period, at admission in the Cardiology Rehabilitation Department (mean time of 10.8 ± 5.7 days after surgery). Red and white blood cells were counted by Sysmex XE-2100 (Dasit; Milan, Italy). Biochemical parameters were measured by UniCel DxC 800 Synchron (Beckman Coulter; Brea, California). They included Creatinine, Sodium and Potassium, C-Reactive Protein, Total Proteins, Alanine Aminotransferase, Aspartate Aminotransferase, Creatin-kinase, Total Cholesterol, Cholesterol HDL, Cholesterol LDL, Triglycerides, Troponin, Ferritin, Transferrin and Fasting Glucose. The TyG index was calculated as TyG = ln [Fasting triglyceride (mg/dl) x Fasting glucose (mg/dl)] / 2).^[25]^ Patients were divided in 2 groups: those affected by known IHD (56 subjects) and those who were not affected by coronary artery disease (44 patients)(control group -CG-) (Table 1).

**Table 1:**
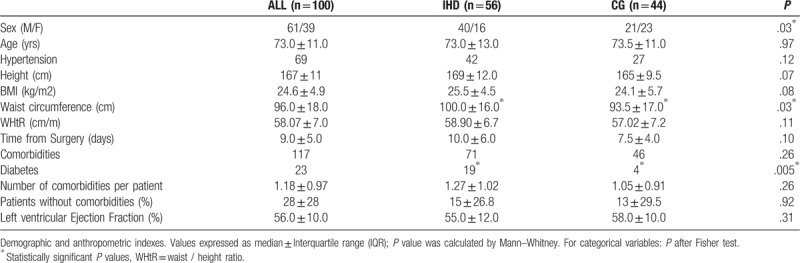
General demographic, anthropometric, and clinical characteristics of individuals with a diagnosis of ischemic heart disease (IHD) and of individuals in the control group (CG).

Patients were classified as affected by IHD if admitted after coronary artery by-pass graft (CABG, alone or combined to valve replacement) or after valve replacement (or valvuloplasty), but with a well-documented history of coronary artery disease, such as previous myocardial infarction or clear atherosclerotic coronary lesions demonstrated at coronary angiography. All the other patients were classified as CG. Both demographic and clinical baseline data are reported in Table 1.

### 2.2 SNARE genotyping

Genomic DNA was isolated from peripheral blood mononuclear cells by phenol-chloroform extraction.

*Stx-1A* and *SNAP25* SNPs were genotyped using the Taqman SNP Genotyping Assays (Applied Biosystems by Life Technologies, Foster City, CA, USA) on an ABI PRISM 7000 Sequence Detection System. Human Pre-Designed Assays (Applied Biosystems by Life Technologies) were used for *SNAP25* rs363050 SNP C_329097_10, *Stx-1A* rs4717806 SNP located in intron 9: C__27872627_10, *Stx-1A* rs2293489 located in *WBSCR22* gene, upstream to 5′UTR *Stx-1A* gene: C__15971044_10.

The *VAMP2* gene 26*bp* Ins/Del polymorphism was genotyped by polymerase chain reaction (PCR). PCR was performed with a GeneAmp PCR System 9700 (Applied Biosystems), using *VAMP2* F-5′-ACAAAGTGCGCCTTATACGC-3′ and *VAMP2* R-5′-GATTTTCCTTGACGACACTC-3′ primers as described in Falbo et al.^[23]^ Amplicons (10 μL) were detected by electrophoresis on a 3% agarose gel.

### 2.3 Statistical analysis

The distribution of biochemical, demographic and clinical parameters was evaluated by Kolmogorov-Smirnov test to assess possible deviations from the Gaussian model. Since the large majority of parameters had a non-normal distribution, the non parametric Mann–Whitney test was adopted to compare the parameters distribution in the groups of IHD vs CG patients.

Binomial logistic regression (forward stepwise analysis) was adopted to evaluate specific biomarkers of IHD risk adjusted for possible bias due to gender, diabetes and circumference waist.

Pearson's chi-square test was performed to compare the distribution of categorical variables in the 2 studied groups as well as to exclude any deviation of SNPs genotype distribution from Hardy–Weinberg equilibrium (HWE). For genotype analyses, chi-square statistics were calculated with 2 degrees of freedom (referred to the three different genotypes). Singular allele frequency association, haplotype analysis distribution and gene interaction score were calculated by SHEsis software http://shesisplus.bio-x.cn/SHEsis.html.^[26,27]^ This analysis allowed to evaluate the association of singular allele on each chromosome giving also information about possible linkage disequilibrium between different alleles. *P*-values were corrected using the Benjamini–Hochberg approach to False Discovery Rate, as provided by SHEsis software.^[28]^

Finally, multiple regression analysis was adopted to evaluate the putative association of biochemical parameters (suitably transformed) with *Stx-1A* genotypes and IHD risk factors.

## 3 Results

### 3.1 Anthropometric and Biochemical characterization of IHD and CG patients

Demographic, anthropometric and clinical variables are reported in Table 1. A higher prevalence of men was reported in IHD (71.4%) than in CG patients (47.7%, *P* = .03). Median waist circumference was higher in IHD than in CG patients (*P* = .05). (T2DM) was diagnosed in 23 patients, nineteen of them being IHD patients (*P* = .005).

Biochemical parameters and Insulin resistance, obtained by calculating the Triglyceride-glucose (TyG) index for each patient^[25]^ are reported in Table 2. Differences in distributions of White blood cell (WBC) count, Creatinin (Crea), AST, Tropinin and Ferritin levels as well as glucose plasma level, lipidic pattern (total cholesterol, LDL and HDL fractions) and TyG index were observed when IHD and CG patients were compared. Binomial logistic forward stepwise regression analysis was used to analyze the possible association of biochemical parameters with different phenotypes of cardiovascular disease (as dependent variable, IHD vs CG) after adjustment for all the other biochemical parameters and for age, gender, diabetes status, and waist circumference.

**Table 2:**
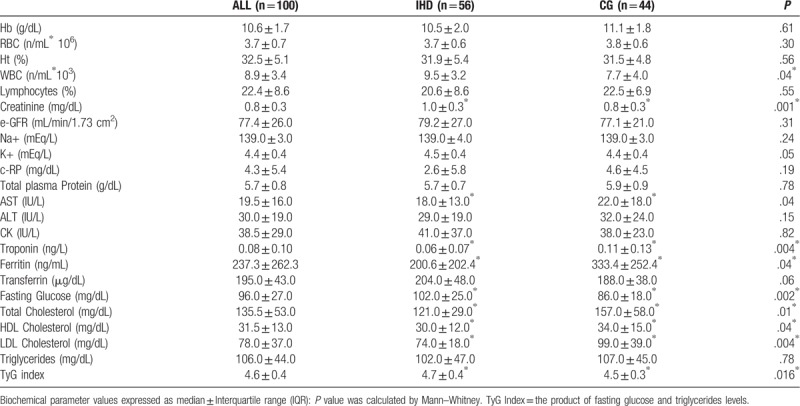
Biochemical parameters distribution in individuals with a diagnosis of ischemic heart disease (IHD) and in individuals in the control group (CG).

A higher fasting glucose level (median ± Interquartile range mg/dL) (102.0 ± 25.0 vs 86.0 ± 18.0; *P* = .002) as well as a higher TyG index (4.7 ± 0.4 vs 4.5 ± 0.3; *P* = .016) were observed in IHD patients compared to controls. These differences retained statistical significance also after imputing as covariate parameters: age, gender, diabetes positivity, waist circumference and cholesterol plasma level (*P* = .03).

### 3.2 Genetic characterization of IHD and CG patients: SNAP25, Stx-1A, VAMP2

All patients were genetically characterized for *SNAP25, Stx-1A*, and *VAMP2* polymorphisms; the genotype distribution resulted in Hardy–Weinberg equilibrium both in the IHD and the CG group. Genetic profiles were then evaluated in both IHD and CG patients. Genotype distribution for *SNAP25, Stx-1A*, and *VAMP2* polymorphisms is reported in Table 3; no genotype association with IHD was observed. However, the comparison done by regression analysis, performed with Shesis software, adjusting for age, gender, diabetes status, waist circumference, total cholesterol, LDL, and HDL fractions as covariates, highlighted a significant association of *Stx-1A rs4717806(A)* and *rs2293489(T)* minor alleles with IHD risk (*Pc* = .02; OR: 2.43, 95%CI:1.1–5.3 and, *Pc* = .02; OR: 2.86, 95%CI:1.3–6.4, respectively) (Table 4) No association was found with the major allele. No statistical skewing was observed as to any other polymorphism analyzed.

**Table 3:**
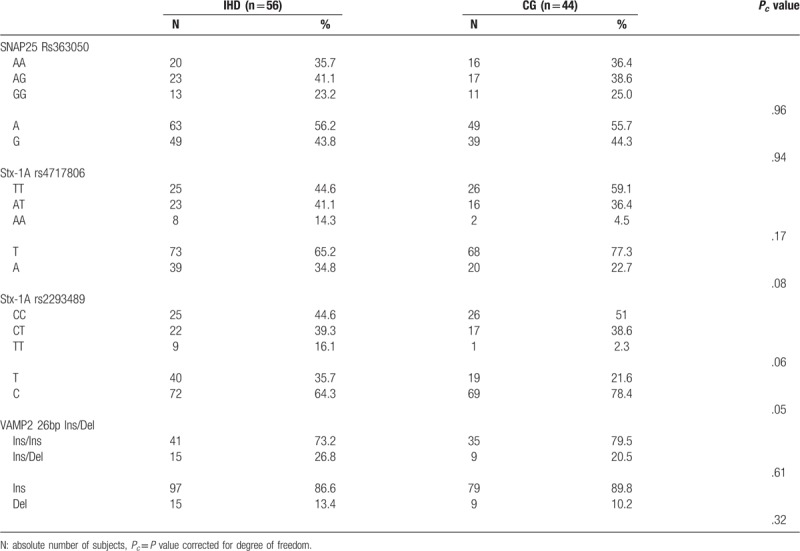
*SNAP25 rs36305, Stx-1A rs4717806*, and *rs2293489*, *VAMP2 26bp* ins/del genotypic and allelic polymorphism distribution in patients with ischemic heart disease (IHD) and in the control group (CG).

**Table 4:**
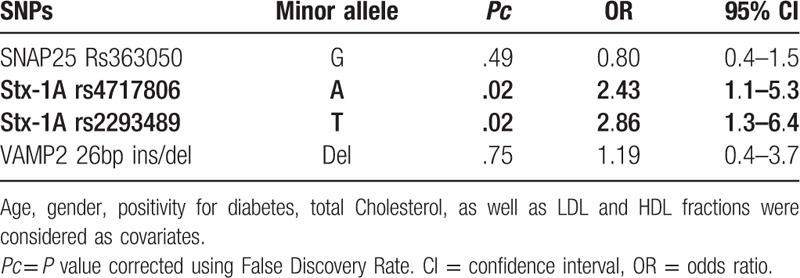
*SNAP25 rs36305, Stx-1A rs4717806*, and *rs2293489, VAMP2 26bp* ins/del, Minor allele effect of association with ischemic heart disease (IHD) vs Control group (CG), calculated by regression analysis model with Shesis software.

Haplotype analysis evidenced a linkage disequilibrium of *rs4717806 and rs2293489* polymorphisms (*r*^2^ = .95) as well as a significant gene-gene interaction (*P* = .04) and a specific haplotype association with ischemic risk. Thus, the *rs4717806 – rs2293489 (A-T)* haplotype was more frequent in IHD (34.8%) than in CG patients (21.5%) (*Pc* = .04; OR:1.94, 95%CI: 1.1–3.7); this result was corroborated by the observation that the complementary haplotype *rs4717806–rs2293489 (T-C)* showed a protective effect (*Pc* = .06 OR:0.53, 95%CI:0.3–1.0) (Table 5).

**Table 5:**
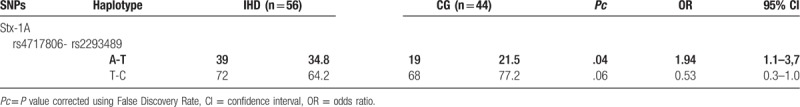
*Stx-1A rs4717806* and *rs2293489* haplotype analysis of distribution in ischemic heart disease (IHD) and Control group (CG) performed by Shesis plus software.

### 3.3 SNAP25, Stx-1A, VAMP2 polymorphisms association with biochemical parameters

The putative association of *SNAP25, Stx-1A, VAMP2* genetic variants with all biochemical parameters in IHD and CG was tested by multiple regression analyses, considering log-transformed biochemical parameters as dependent variable and presence of coronary artery disease, age, gender, waist circumference, presence of diabetes as predictors. The logarithmic transformation allowed to reduce the degree of skewness of the distribution of biochemical parameters.

**Figure 1:**
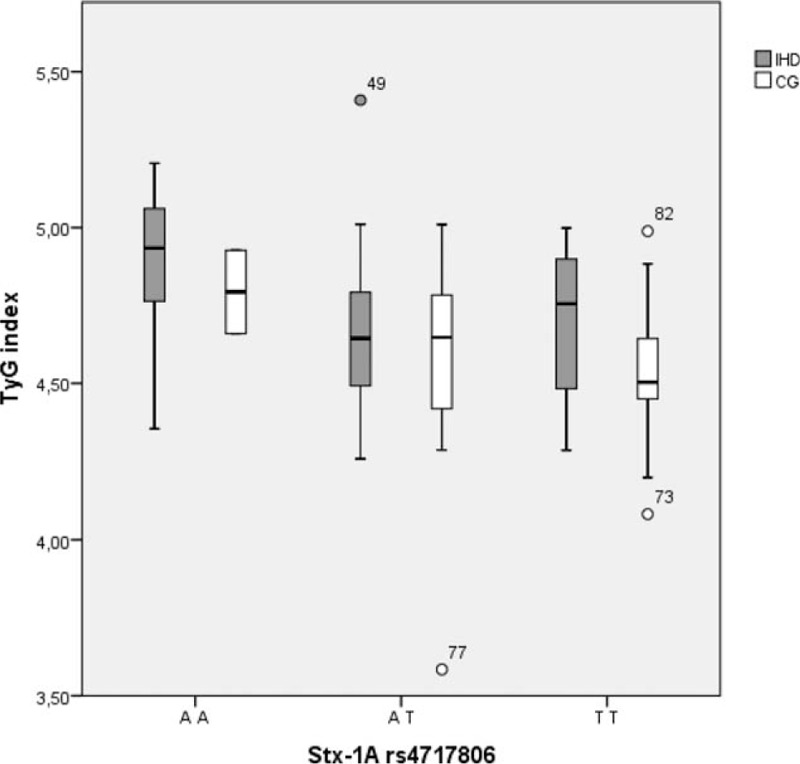
Triglyceride-glucose (TyG) index distribution in relationship with *Stx-1A rs4717806* genotype in 56 patients affected by ischemic heart disease (IHD) (grey boxes) and in 44 subjects of control group (CG) (white boxes). Outline values are represented by code number.

Results showed that the *Stx-1A rs4717806(TT)* genotype was associated with a protective effect on both Triglyceride levels and TyG index compared to the *(AA)* genotype (β = −0.29, SE = 0.15, *P* = .05) and (β = −0.05, SE = 0.02, *P* = .01), respectively. This genotype was associated with the TyG Index alone in the subsample of IHD patients (β = −0.04, SE = 0.02, *P* = .036) (Fig. 1). No significant association did emerge as to *Stx-1A rs2293489* polymorphism. No other association emerged in any of the other parameters evaluated.

## 4 Discussion

The primary finding of this study is the characterization of a significant association between the *Stx-1A* polymorphisms *rs4717806(A*) and the *rs2293489(T)* alleles and IHD development, both as single minor alleles, and as haplotype. Moreover, the correlation analysis of SNARE genetic polymorphism with metabolic biochemical risk factors for IHD evidenced the presence of a significant higher level of TyG index in IHD patients carrying the *Stx-1A rs4717806(A)*, but not the *Stx-1A rs2293489(T)* allele, after adjustment for possible confounding factors. This observation suggests that *rs4717806(A)* involvement in IHD is stronger than involvement of *Stx-1A rs2293489(T)* allele, whose association with IHD may be due to a linkage disequilibrium with *rs4717806(A)*. It is noteworthy that STX1A rs4717806 (AA) genotype was associated with a lower protein expression with a tendency for gene dose effect.^[19]^

TyG index is a useful surrogate marker for insulin resistance,^[25]^ which is independently associated with the presence of coronary artery atherosclerosis;^[29]^ notably, this parameter was recently shown to be an important risk factor for IHD.^[30,31]^ Insulin resistance leads to a relative reduction of insulin action, then to hyperglycemia and consequently to higher production of insulin by pancreatic β-cells.

Insulin exocytosis depends on the intracellular storing of insulin within vesicles, vesicle trafficking and fusion to the membrane of pancreatic β-cells.^[32,33]^ SNARE proteins constitute the ‘core complex’ that regulates vesicle trafficking and fusion in β–cells.^[34]^ Within these proteins, Stx-1A has been shown to bind and regulate calcium channels and voltage gated Kþ channels of the pancreatic β–cell.^[35,36]^ Recently, in transgenic mice for Stx-1A, it was demonstrated that subtle fluctuations in the expression of this protein determine changes in insulin secretion, ultimately resulting in glucose intolerance.^[37]^ The *Stx-1A* gene has been mapped to chromosome 7q11. A quantitative trait locus for fasting glucose has been found linked to 7q in Europeans,^[38]^ thus adding confirmatory evidence that this region may contain gene or genes with an important impact on insulin and glucose metabolism regulation.^[39]^

Evidence here reported leads to assume a putative role of *Stx-1A rs4717806* in IHD, possibly due to its influence in insulin-dependent glucose metabolism and therefore also in altered lipids pathways, which are well-known risk factors for cardiovascular disease.^[40]^ We cannot nevertheless exclude that the correlation of Stx-1A with IHD is the effect of a lower efficacy of K_ATP_ channel, resulting in higher risk for atherosclerosis.^[11]^

So far Stx-1A protein has been reported also to be able to regulate myocardial injury-related signaling pathways such as K_ATP_ channels and calcium channels.^[14,15]^ Increased Stx-1A expression has been suggested to participate in cell salvage or repair, as a consequence of its ability to mediate neurotransmitter release and plasma membrane recycling, thereby exerting protection.^[18]^ A lower expression of this protein, thus, could reduce hypoxia/reoxygenation-induced cardiomyocyte apoptosis and cell viability, resulting in a reduced degree of cardioprotection.^[12]^

Notably, even if our results will need to be replicated in larger cohorts of IHD patients, they are consistent with evidence reported by an important Genome-Wide Association study (GWAS), which showed a strong association of 7q11 region with Triglyceride alterations.^[41]^*Stx-1A* maps in a region 70kb close to one of the genes suggested by GWAS: that is MLX-interacting protein-like (MLXIPL), significantly associated with Coronary heart disease.^[42]^ An in-depth evaluation of the *Stx-1A* SNPs influence in cardiologic risk is warranted to confirm the involvement of these SNPs in IHD.

## 5 Conclusions

Our results suggest a role of *Stx-1A rs4717806* SNP in IHD, possibly due to its influence in Stx-1A expression and, at cascade, to insulin secretion and to glucose dependent metabolism.

This study may be considered a preliminary investigation; if confirmed by further studies, these results could help in identifying those individuals in whom strong efforts to prevent metabolic disorders and reduce cardiological risk is needed.

## Author contributions

**Conceptualization:** Franca Rosa Guerini, Andrea Saul Costa, Mario Clerici, Vittorio Racca.

**Data curation:** Franca Rosa Guerini, Enrico Ripamonti, Vittorio Racca.

**Formal analysis:** Enrico Ripamonti.

**Funding acquisition:** Mario Clerici, Vittorio Racca.

**Investigation:** Andrea Saul Costa, Milena Zanzottera, Cristina Agliardi, Elisabetta Bolognesi.

**Project administration:** Franca Rosa Guerini, Mario Clerici, Vittorio Racca.

**Resources:** Vittorio Racca, Mario Clerici.

**Supervision:** Franca Rosa Guerini, Vittorio Racca.

**Validation:** Franca Rosa Guerini, Elisabetta Bolognesi.

**Visualization:** Franca Rosa Guerini, Andrea Saul Costa, Cristina Agliardi, Vittorio Racca.

**Writing – original draft:** Franca Rosa Guerini, Enrico Ripamonti, Mario Clerici, Vittorio Racca.

Franca Rosa Guerini orcid: 0000-0001-9461-5927.

Franca Rosa Guerini orcid: 0000-0001-9461-5927.
